# Regenerative medicine in lung diseases: A systematic review

**DOI:** 10.3389/fvets.2023.1115708

**Published:** 2023-01-17

**Authors:** Neža Adamič, Modest Vengust

**Affiliations:** Faculty of Veterinary Medicine, University of Ljubljana, Ljubljana, Slovenia

**Keywords:** lung, regenerative medicine, veterinary medicine, lung diseases, cellular therapies, biomedical engineering

## Abstract

Regenerative medicine has opened the door to the exploration of new therapeutic methods for the treatment of various diseases, especially those associated with local or general disregulation of the immune system. In pulmonary diseases, new therapeutic strategies have emerged that are aimed at restoring functional lung tissue rather than alleviating symptoms. These strategies focus on tissue regeneration using stem cells and/or their derivatives or replacement of dysfunctional tissue using biomedical engineering. Animal health can directly benefit from regenerative therapy strategies and also serve as a translational experimental model for human disease. Several clinical trials have been conducted to evaluate the effects of cellular treatment on inflammatory lung disease in animals. Data reported to date show several beneficial effects in *ex vivo* and *in vivo* models; however, our understanding of the mechanisms that regenerative therapies exert on diseased tissues remains incomplete.

## Introduction

Several chronic respiratory diseases in humans and animals remain incurable. Treatments have been relatively successful in relieving some symptoms, but they all ultimately lead to a poorer quality of life and are one of the leading causes of death worldwide (1). Intense and persistent inflammation leads to loss of functional tissue and pulmonary tissue remodeling, which in turn leads to loss of respiratory function. Over time, lung tissue changes are so severe that euthanasia is required in animals or lung transplantation is the only viable option to prolong life in humans (2–9).

The lung has an exceptional ability to respond and regenerate after the tissue injury (7, 9, 10). However, regeneration of lung tissue can often lead to pathological tissue remodeling and subsequent impairment of lung function (10). These changes in the lung can potentially be reversed through regenerative medicine in the form of cellular therapy, extracellular vesicle therapy (ECV), or even tissue engineering (4, 5, 7, 9–12). Several animal studies addressed regenerative therapeutic modalities in the lung in experimental models (13–38) and in clinical trials (39–41) (Table 1).

**Table 1:** List of selected references reporting the use of regenerative treatments for respiratory diseases in animals.

**Disease**	**Species**	**Therapeutic strategy**	**Treatment outcomes**	**References**
Acute lung injury	Mice	Bone marrow-derived mesenchymal stem cells	Treatment with intrapulmonary MSC markedly decreases the severity of endotoxin-induced acute lung injury and improved survival in mice	Gupta et al. (13)
	Rabbit	Bone marrow-derived mesenchymal stem cells	Decreased pro-inflammatory cytokines, increased anti-inflammatory cytokines, decreased lung water mass fraction, and ameliorated systemic inflammatory response.	Zhu et al. (15) Chen et al. (16)
	Mice	Human Umbilical cord blood-derived mesenchymal stem cells	Down-modulated inflammatory process and enhanced bacterial clearance.	Kim et al. (17) Sun et al. (19)
	Rats	Human umbilical cord blood-derived mesenchymal stem cells	Reduced systemic inflammation and attenuated ALI	Li et al. (21)
	Sheep	Human bone marrow-derived mesenchymal stem cells	Reduced severity of ALI	Asmussen et al. (22)
	Dogs	Human Umbilical cord blood-derived mesenchymal stem cells	Reduced lung injury.	Hao et al. (33)
	Pigs	Extracellular vesicle therapy	Attenuated influenza virus-induced acute lung injury.	Khatri et al. (34)
	Rats	Extracellular vesicle therapy	Alleviated lung injury and pulmonary fibrosis.	Gao et al. (42)
Acute respiratory distress syndrome	Sheep	Human bone marrow-derived mesenchymal stem cells	Ameliorated inflammation.	Rojas et al. (25) Sadeghian Chaleshtori et al. (37)
	Sheep	Adipose-derived mesenchymal stem cells	Attenuated pulmonary microvascular hyperpermeability.	Ihara et al. (32)
	Sheep	Bone marrow-derived multipotent adult progenitor cells	Recovered arterial oxygenation.	Cardenes et al. (36)
Asthma	Mice	Human bone marrow-derived mesenchymal stem cells	Decreased chronic inflammation	Bonfield et al. (14) Lee et al. (18) Mohammadian et al. (27) Cruz et al. (26)
	Mice	Adipose-derived mesenchymal stem cells	Ameliorated allergic airway inflammation.	Cho et al. (23) Mariñas-Pardo et al. (24) Dai et al. (29) Dai et al. (30)
	Horses	Bone marrow-derived mononuclear cells	Reduced airway inflammation	Barussi et al. (39)
	Mice	Bone marrow, adipose, and lung tissue-derived mesenchymal stromal cells	Reduced airway inflammation and remodeling and improved lung function.	Abreu et al. (28)
	Cats	Adipose-derived mesenchymal stem cells	Delayed effect in reducing airway inflammation, airway hyper-responsiveness and remodeling.	Trzil et al. (40)
	Mice	Human adipose-derived mesenchymal stem cells and their extracellular vesicles	Reduced inflammation and modulated airway remodeling.	de Castro et al. (31)
	Horses	Adipose-derived mesenchymal stem cells	Limited short-term anti-inflammatory effects and long-term stability of clinical signs	Adamič et al. (41)
Lung emphysema	Sheep	Autologous lung-derived mesenchymal stem cell	Ameliorated lung perfusion	Ingenito et al. (20)

Therapeutic strategies and treatment outcomes are also listed.

Although inflammation is the reason for damage to the airways, it is also critical for initiating tissue regeneration and restoration. Inflammatory cells flooding the airways are important for phagocytosis and for stimulating resident progenitor cells through secreted cytokines and growth factors. Some resident cell populations do not appear to exist in a healthy lung, but emerge only in response to lung injury. A more detailed knowledge of this relationship will likely enable new therapeutic options to stimulate lung regeneration and self-repair (43).

## Data sources and searches

An online literature search was performed using the PubMed^®^ (U.S. National Library of Medicine and National Institutes of Health) search engine (https://pubmed.ncbi.nlm.nih.gov/), evaluating reports from January 1, 1990, to October 31, 2022. Reference lists of relevant articles were also reviewed to find additional studies.

## Cellular therapy

Regenerative cell therapy is currently the most widely used method for stimulating the regeneration of damaged tissue, in which stem cells (SC) play a leading role. Stem cells are undifferentiated cells capable of self-renewal and transformation into other cell types (44). Traditionally, the therapeutic effect of SC has been associated with their migration to the affected area and their ability to replace damaged tissue (45). However, later discoveries recognized their complex immunomodulatory role through interaction with local cells of the immune system and paracrine signaling (46, 47). Currently, two ways of their potential use for therapeutic purposes are being investigated: (1) induction of endogenous differentiation and mobilization of resident progenitor cells and (2) *ex vivo* (exogenous) cultivation of SC and their application in patients (4, 11, 48, 49). The former is mainly related to tissue regeneration and repair through activation of resident cells (43, 48), while the latter is mainly associated with paracrine action and immunomodulatory effects (4).

Heterogeneous endogenous stem cells [cells capable of long-term self-renewal and differentiation into other progenitor cells or tissue-specific cells (4)] and progenitor cells [tissue-specific cells capable of differentiation into specific cell types, but are not capable of self-renewal or are capable of self-renewal only in the relatively short term (4)] of the lung, located in different regions of the airway, are capable of self-renewal and of forming one or more mature cell types, allowing local maintenance of epithelial integrity and repair of damage (4, 10, 50). They reside in their unique microenvironmental niches that allow them to maintain their progenitor properties and differentiate into different cell types (10). Several distinct populations of stem and progenitor cells are present in the airways, which can differentiate into different airway cell types (4, 10, 50).

Basal epithelial cells represent a population of stem/progenitor cells from which Club cells (formerly known as Clara cells) and ciliated cells can develop (51, 52). They may also serve as progenitors for multiciliated and goblet cells (10). Submucosal glandular progenitor cells are another group of cells capable of regenerating submucosal glandular tubules, ducts, and surface epithelium (10, 53). Neuroendocrine cells of the lung can also function as progenitor cells that differentiate into Club cells and ciliated cells upon injury (10, 54). Type 2 alveolar cells are critical for surfactant C production and secretion, but are also considered alveolar progenitor cells. They can self-renew and/or differentiate into type 1 alveolar cells, which are responsible for gas exchange (10). Differentiation, proliferation and expansion of type 2 alveolar cells after tissue injury is protracted and takes several months (50).

Resident stem cells, which are thought to share several properties with bone marrow-derived mesenchymal stem cells (BM-MSC), have also been found in the lung (20, 55–57). They are currently referred to as lung mesenchymal stem cells or lung mesenchymal stromal cells (L-MSC). Their potential physiological or pathophysiological functions are not yet known. Similar to BM-MSC, L-MSC secrete immunosuppressive molecules and therefore may influence the course of inflammation, tissue injury and repair (58, 59).

Attempts have also been made to derive the phenotype of structural lung cells for pulmonary vascular regeneration from adipose or bone marrow tissue or from embryonic SC. Despite the ability of SC to differentiate into lung cell types, results of such studies remain controversial because of inadequately derived or described methods (4).

The therapeutic potential of exogenous SC has been repeatedly noted in relation to their immunomodulatory effects. Their complex immunomodulatory role results from their interaction with local immune cells and paracrine signaling, leading to a reduction in proinflammatory stimulus and thus less tissue damage (46, 47, 60–63). Most research on SC therapies has focused on inflammatory airway diseases where conventional treatments have been unsuccessful, and they have been found to have several beneficial effects (36, 64–67). Various cell sources (e.g., BM-MSC, adipose-derived stem cells, embryonic stem cells, umbilical cord blood-derived mesenchymal stem cells), dosages, and delivery methods have been investigated to maximize the potential of their therapeutic use. However, there is not yet sufficient evidence to formulate precise guidelines for clinical use.

## Tissue engineering

Pathologic changes in diseased lungs may progress to the point where cell therapy and stimulation of tissue regeneration alone are insufficient and tissue replacement is required to restore lung function. Suitable lung donors are not always available, or lung transplantation is contraindicated (68); therefore, *in vitro*-grown tissue may bridge the time to lung transplantation or serve as a definitive therapeutic modality.

Tissue engineering techniques are still insufficiently developed. The lung is composed of more than 40 different cell types that form a complex three-dimensional (3D) anatomic architecture (69). Generating lungs *in vitro* and mimicking their function is a major challenge that requires a high degree of cell specialization and complex tissue architecture (5, 48, 70, 71). They must provide a variety of organ functions, such as the diversity of airway cell types, the defense mechanisms that protect the upper airways (e.g., secretion of specifically composed mucus and active ciliary apparatus), and the coupling of the alveolar space with the surrounding systemic and pulmonary vasculature to ensure effective tissue perfusion and gas exchange (72).

Most preclinical studies have used biologically derived models or synthetic scaffolds seeded with an appropriate cell source to regenerate functional lung tissue (5, 7). Hybrid scaffolds combining biological materials (extracellular matrix (ECM) components) with synthetic scaffolds currently appear to have the greatest potential. These scaffolds are then seeded with autologous or allogeneic cells to generate functional tissue generation (5). An important advantage of using allogenic cells is the reduction of immunologic complications and tissue rejection (7, 12, 48). In this way, a miniaturized and simplified version of an organ can be produced in the laboratory, called an organoid. This is a 3D structure that replicates the microanatomy of the desired organ. The formation of organoids relies on the self-assembly of cells derived from adult tissues, embryonic stem cells, or induced pluripotent stem cells (70, 71, 73, 74).

Because they represent the overall architecture of the lung, organoids are important models for studying various physiological processes in the airway microenvironment and the effects of various effectors on airway tissue structure, including infectious agents and/or new therapeutic modalities. This is particularly important because the cellular and molecular response to chemical and physical signals *in vivo* and the properties of gene expression can be obscured or lost in more commonly used *in vitro* 2D cell culture systems (73, 74). Lung organoids are broadly divided into proximal lung organoids (containing cells that mimic the conducting airways), distal lung organoids (subsuming the alveoli), or proximal-distal organoids (74).

The creation of a functional epithelial tissue appropriately connected to the vascular component is particularly important for the future development of therapeutically beneficial engineered pulmonary tissues. A more ambitious model of tissue engineering is based on decellularization of the original organ, in which all cells and cellular materials are removed from the entire lung, resulting in an intact three-dimensional scaffold. This represents the innate ECM, preserving the natural structure of the airways and blood vessels, providing an optimal platform for transplantation of lung cells (48, 74). Lung ECM (collagen and elastic fibers enriched in proteoglycans, glycosaminoglycans, and fibronectin) not only provides a sophisticated scaffold for potential lung organogenesis, but also combines biochemical and mechanical signals that further guide SC behavior during lung re-development and regeneration (74). To generate functional lung tissue *ex vivo*, one would need to define more than 40 different cell types and perhaps hundreds to thousands of different cell subtypes (5).

Nichols et al. (35) transplanted a bioengineered porcine lung, which was generated using autologous cells. The bioengineered lungs successfully formed alveolar tissue and were ventilated, well vascularized, and developed a microbiome similar to that of the natural lung. The authors also noted no evidence of graft rejection (35). However, Yanagiya et al. (38) reported marked bullous changes in the transplanted tissue of bioengineered lungs when they examined unilateral transplantation of porcine lungs generated from autologous cells. They also reported comparable oxygen exchange between the bioengineered lung transplant group and the allograft recipient group, whereas CO_2_ exchange was significantly lower in the bioengineered lung transplant group than in the allograft group (38).

Airway anatomy and physiology are highly species-dependent, making it necessary to create species-specific models. In a recent review of mammalian lung organoids, Archer et al. (72) highlighted that the cells lining the bronchiolar or more distal part of the tracheobronchial tree differ considerably between species in terms of their abundance, the cell types present, the ultrastructural features of these cells in adult animals, and the secretory products they produce (72). Mouse models, for example, are not particularly well suited for studying human respiratory diseases. On the other hand, sheep lungs are most commonly used as models for human lungs because of their anatomy and the uniform distribution of differentiated cells at a given age of maturity. These elements make sheep a valuable model for human respiratory physiology and disease (72).

## Cell-free therapeutical strategies

Extracellular vesicles are membrane-protected carriers of many substances, including microRNA (miRNA), messenger RNA, proteins, and mitochondria. Extracellular vesicles are broadly classified into exosomes (vesicles of endocytotic origin with a diameter of 30–150 nm, surrounded by a plasma membrane), microvesicles (diameter of 100–1,000 nm, not of endocytotic origin), and apoptotic bodies [diameter of 50 nm−5 μm; they are released by apoptotic cells during membrane budding (blebbing)] (75). The use of ECV offers several important advantages over cell therapy. Due to their smaller size, ECV can penetrate deeper into the airways and potentially be delivered by inhalation techniques (76). In addition, their membrane envelope makes them stable in tissues and body fluids. They also have low immunogenicity and toxicity compared to cell therapies (77, 78). A major obstacle to the therapeutic use of ECV is the lack of standardized methods for isolation and purification of ECV. The lack of standardized methods for isolating exosomes means that exosomes cannot be separated from other ECV of similar size. There is also a lack of standardization of methods for measuring ECV purity (47, 79). In this context, it is advisable to use the generic term “extracellular vesicle” when using ECV therapeutically and to avoid nominal categorization into subtypes. If the name of a single subspecies is used, extraction and selection must be precisely defined.

Confirmation of the functionality of ECV therapy requires that the therapeutic effect occurs without intercellular contact and that this is not achieved by ECV-unrelated soluble paracrine factors (80). Extracellular vesicles are involved in several intercellular signaling pathways, making them critical molecular messengers in various processes responsible for normal homeostasis and disease development. In the regeneration process, they also influence the response of stem/progenitor cells and other cells within their niche (78).

Numerous studies have demonstrated the benefits of systemic administration of ECV in mitigating allergic airway hyperreactivity and resulting inflammation and tissue remodeling (26, 31). Extracellular vesicle treatment has been shown to be beneficial in the treatment of lung injury and pulmonary fibrosis in rats. After intratracheal administration, there was a reduction in apoptosis and necrosis of type 2 alveolar epithelial cells and alleviation of lung injury. Extracellular vesicles decreased reactive oxygen species levels and inflammation in the airways. The authors were able to attribute some of the beneficial effects to a specific miRNA, let-7d-5p (42). Antounians et al. (81) also attributed the therapeutic effects of ECV to miRNA when they investigated its influence on the regenerative capacity of undeveloped fetal lungs in an experimental rodent model. Following ECV treatment, enhanced morphogenesis and alveolarization, restoration of lung tissue homeostasis, and differentiation of epithelial cells and fibroblasts were observed in association with the release of RNA cargo (81). In addition, ECV treatment may limit viral respiratory infections by affecting viral replication and virus-induced apoptosis in lung epithelial cells, which is also thought to depend on the transfer of RNA from ECV to epithelial cells (34).

In addition to the cell-free regenerative medicine options described above, several indirect therapeutic options have been described to stimulate local cells and tissue regeneration in the airways. For example, all-trans-retinoic acid, a derivative of vitamin A (retinol), has been described as a possible candidate to promote alveologenesis (48, 82, 83). It is also suggested that nanoparticles of integrins may influence the regeneration of collapsed alveoli (84). Another area of research is regenerative photobiostimulation, which aims to stimulate resident stem cells with electromagnetic radiation to trigger growth factor production, inhibition of inflammation, and stimulation of angiogenesis (85).

## Most probable therapeutic application in animals

Benefits of cell treatments have been reported for the treatment of asthma; experimentally in mouse models (14, 18, 23, 24, 26–31) and animals with natural asthma, such as cats (40) and horses (39, 41). The treatment effects of SC, identified in preclinical studies of asthma treatment, are related to the reduction of airway inflammation through the regulation of inflammatory cytokines. The results of these studies differ in terms of cytokine expression and translation, but all consistently reported a reduction in airway inflammation. The influence of SC on tissue remodeling may play the critical role in the treatment of asthma (18, 24, 26, 31, 86, 87).

Ingenito et al. (20) investigated the effect of autologous L-MSC on experimentally induced lung emphysema in sheep to evaluate their ability to regenerate functional tissue. Animals received endoscopically either cellularized biological scaffolds or scaffolds alone. At four-week follow-up, no immune response to the grafts was detected, but significant improvement in tissue mass (in terms of increased cellularity and extracellular matrix content) and lung perfusion was observed in sheep receiving L-MSC compared with the control group. Detection of labeled L-MSC in the alveolar septum and peribronchial interstitium was also reported. L-MSC therefore have the potential for regeneration of emphysematous lungs (20).

Treatment with SC also significantly affects inflammatory responses and lung tissue regeneration in acute lung injury (ALI) and acute respiratory distress syndrome (ARDS) (36, 88–91). Aside from symptomatic therapy, no specific treatment for these diseases have been defined that would substantially improve short- and long-term outcomes. Positive effects in terms of reducing pulmonary edema and inflammation and improving gas exchange have been reported after cell treatment in experimentally induced ARDS in sheep (22, 25, 32, 36, 37). Currently, research on the effect of SC on the treatment of ARDS caused by respiratory viruses is particularly relevant due to the COVID-19 pandemic (67, 90, 92). Reductions in oxidative stress and inflammation and resulting lung injury and mortality following treatment with SC have been observed in mice (13, 17, 19, 21), rabbits (15, 16) and dogs (33) with experimentally induced lung injury.

In addition to cell therapy, treatment with ECV has also successfully treated acute airway inflammation caused by viral infection. Khatri et al. (34) investigated the effects of intratracheally administered ECV on influenza virus-induced acute lung injury in pigs. ECV treatment significantly reduced viral secretion (detected in nasal swabs), viral replication in the lungs, and virus-induced inflammatory cytokine formation in the lungs of infected pigs 12 h after viral infection. The authors concluded that intratracheal treatment with ECV attenuates influenza virus-induced ALI in pigs (34).

## Conclusions

Further evidence from appropriately designed clinical trials is needed before regenerative therapy is considered an accepted therapeutic modality in respiratory medicine. To date, the use of SC or ECV for the treatment of respiratory disease has consistently been described as relatively safe after local and systemic application. Apart from mild local reactions after administration of cells of allogeneic origin, no severe adverse events have been observed (41, 47, 93–96). The interaction between SC/ECV and the immune system may also provide better insight into the pathophysiology of immune system dysregulation in the respiratory system.

It is also important to focus on a detailed understanding of the functional heterogeneity of each cell type in the respiratory system and the development of protocols for targeted cell differentiation and maturation (70). This is particularly true for tissue engineering, which is less explored compared to SC and ECV due to its anatomical and functional complexity. The creation of a functional epithelial tissue suitably linked to the vascular component is particularly important for the future development of respiratory physiology and medicine.

## Author contributions

All authors listed have made a substantial, direct, and intellectual contribution to the work and approved it for publication.
